# Algorithmic crime prevention. From abstract police to precision policing

**DOI:** 10.1080/10439463.2024.2326516

**Published:** 2024-03-06

**Authors:** Simon Egbert, Elena Esposito

**Affiliations:** a Faculty of Sociology, Bielefeld University, Bielefeld, Germany; b Department of Political and Social Sciences, University of Bologna, Bologna, Italy

**Keywords:** Abstract police, predictive policing, crime prevention, precision policing

## Abstract

The growing digitisation in our society also affects policing, which tends to make use of increasingly refined algorithmic tools based on abstract technologies. But the abstraction of technology, we argue, does not necessarily entail an increase in abstraction of police work. This paper contrasts the ‘abstract police’ debate with an analysis of police practices that use digital technologies to achieve greater precision. While the notion of abstract police assumes that computerisation distances police officers from their community, our empirical investigation of a geo-analysis unit in a German Land Office of Criminal Investigation shows that the adoption of abstract procedures does not by itself imply a detachment from local reference and community contact. What we call contextual reference can be productively combined with the impersonality and anonymity of algorithmic procedures, leading also to more effective and focused forms of collaboration with local entities. On the basis of our empirical results, we suggest a more nuanced understanding of the digitalisation of police work. Rather than leading to a progressive estrangement from the community of reference, the use of digital techniques can enable experimentation with innovative forms of ‘precision policing’, particularly in the field of crime prevention.

## Introduction

In recent years the use of algorithms by the police has increased significantly, triggering a lively debate about predictive policing and its positive and negative consequences (Edwards 2017, Ferguson 2017, Bennett Moses and Chan 2018, Kaufmann *et al.* 2019, Brayne 2021, Egbert and Leese 2021, Hälterlein 2021, Sandhu and Fussey 2021). During the same period the discussion on ‘abstract police’ emerged, which also reflects on the consequences of the digitalisation of police work (Seidensticker and Bode 2022, Terpstra and Salet 2022, Terpstra, Fyfe, *et al.* 2022). Abstract police is understood as a tendency of the police to become increasingly rigid, formalised, delocalised and distant from citizens (Terpstra *et al.* 2019, Terpstra, Salet, *et al.* 2022a, 2022b). Are these two trends related to each other, how, and with what consequences? Up to now there is no empirically grounded reflection available on the connection between algorithmic policing and the growing abstraction of police work. In this article we intend to provide it, based on empirical findings on a geo-analysis unit of a German State Criminal Police Office. Our hypothesis is that the joint analysis of the debate on digital policing and abstract policing brings out interesting potential for studying the innovative use of digital techniques by the police with the aim of making policing more precise, particularly in the field of prevention.

Our argumentation is structured as follows: First we present the recent debate on abstract police and describe its core message, especially with reference to digitalisation. In a second step we describe the evolution of the use of algorithms in policing, moving from the initial idea of predictive policing targeting specific individuals and groups to an idea of ‘precision policing’ aimed at developing more robust and effective community engagement of police forces. We then showcase our empirical case study, focussing on the practices of a geo-analysis unit of a German federal state police office, where algorithms are used to develop place-based policing approaches, mostly focussing on crime prevention strategies. In the subsequent section, we discuss the relevant findings referring to three projects of the unit we studied and connect the notion of abstract police with the recent debate about precision policing. We close with a concluding section summarising our argument and pointing to possible future research directions.

## Abstract police

In 2019, Jan Terpstra, Nicholas F. Fyfe and Renze Salet (Terpstra *et al.* 2019) started a debate on recent trends in police organisations and introduced the notion of ‘abstract police’. Referring to the results of their comparative study of police reforms in the Netherlands and in Scotland, they observed a fundamental organisational shift in policing, which is getting increasingly abstract in its internal as well as in its external relations. The notion of abstract police indicates that ‘the police have become more at a distance, more impersonal and formal, less direct, and more decontextualized’ (Terpstra *et al.* 2019, p. 340). In social relations and practices of police forces, the role of human individuals is reduced and increasingly replaced by ‘systems, system knowledge, communication at a distance’ (Terpstra, Salet, *et al.* 2022b, p. 14). Both in the Dutch and in the Scottish police, major reforms introduced a significant move towards centralisation, resulting in larger work areas for police officers and increasing alienation from ‘their’ patrol areas. In addition, greater formalisation and fragmentation of the police was implemented, as most operations needed to follow more closely given procedural guidelines, and local idiosyncrasies tended to be disregarded.

Discussing the factors contributing to the trend towards increasing abstraction, the authors put special emphasis on the growing reliance on IT-technology and computers (Terpstra, Fyfe, *et al.* 2022, Terpstra, Salet, *et al.* 2022b, pp. 3–4). Echoing the seminal analysis of Ericson and Haggerty (1997), Terpstra *et al.* (2019, p. 350) argue that police officers become ‘more dependent on “system information” at the cost of direct and “personal” information’, devaluating the craft of policing and the discretionary power of police officers (see also Terpstra, Salet, *et al.* 2022b, p. 19). The rise of intelligence-led policing (see e.g. Ratcliffe 2016) highlights the necessity of collecting, processing and analysing information, leading to growing reliance on specialists and a strong focus on intelligence, while devaluating ‘the value and importance of direct, personal and often informal knowledge of police officers’ (Terpstra *et al.* 2019, p. 351, see also Gundhus *et al.* 2022, 2023).

Against this backdrop, Terpstra and Salet (2022) discuss the role of predictive policing, especially referring to the Dutch predictive policing software CAS (Crime Anticipation System), which provides a risk map to better allocate patrol officers to areas where the risk of crime is deemed to be higher. This map-based algorithmically generated information, they claim, enters into competition with the knowledge of community officers and contributes to turning community policing to an office job, distancing community officers from ‘their’ communities (Terpstra and Salet 2022, p. 91).^1^ The connection of abstract police and predictive policing is also drawn by Seidensticker and Bode (2022), who developed themselves a crime prediction tool for the North Rhine-Westphalian police in Germany (Landeskriminalamt NRW 2018 Seidensticker 2021, Bode and Stoffel 2023,). They argue that police work informed by crime prediction software ‘is no longer based on traditional patterns of street-level police officers’ (Seidensticker and Bode 2022, p. 175), since mathematical algorithms decide where officers shall patrol. In their view, system information becomes more important than personal information, ultimately making policing more abstract.

Terpstra *et al.* (2019, p. 343) underline that their notion of abstract police should be considered an ‘ideal-typical concept’ abstracting from empirical social reality (Terpstra, Fyfe, *et al.*, 2022, p. 6), and that further empirical studies have to show where refinements are necessary. The following analysis should also be read against this background.

## Precision policing

The debate on abstract police seems to be vitiated by the overlapping between two different distinctions: the distinction *anonymous/personal* and the distinction *acontextual/contextual*. The notion of abstractness brings both distinctions together, although the conditions and consequences of the two distinctions are significantly different. This creates analytical frictions, which, we argue, impair the empirical analysis of digitalised policing in general and predictive policing in specific.

The first distinction, anonymous/personal, concerns whether or not police officers have direct contacts with citizens and/or know them in person. The second distinction, acontextual/contextual, concerns the presence or absence of a local reference that takes into account the specificity of each context – in terms of space, local structures, relationships inside the community. Although connected, the two distinctions do not overlap: police activity can be impersonal and contextual, or personal and non-local. Both distinctions have different assumptions and consequences, and, above all, a different connection with the digitisation of police work – with the use of computers or with the recent algorithmic techniques employed, for example, in predictive policing. We argue that the lack of differentiation between these dimensions leads to a simplified assessment of the relationship between abstract police and digitalisation.

The evolution of digital policing techniques in the U.S. can be regarded as an indirect demonstration of the difference between the two distinctions anonymous/personal and acontextual/local. The company PredPol, which produced what for several years has been the most widely used predictive policing algorithm in the United States (Ferguson 2017, p. 65), recently changed its name to Geolitica (Geolitica 2021) to highlight the distinction between individual prediction and a local-reference-oriented forecasting approach. PredPol’s controversial original design aimed at ‘prevent(ing) (…) crimes from occurring’ (PredPol 2020) in targeted areas where the risk of crime in the coming days is considered to be the highest (Brayne 2021, pp. 70–71). Precisely in the sense of abstract police, these techniques substituted computerised procedures for direct personal knowledge of place and areas by police officers. Widespread criticism of this type of prediction style (e.g. Lum and Isaac 2016, O’Neil 2016, Ferguson 2017, Brayne 2021) has recently prompted the company to change its name, even dropping the explicit reference to prediction. In fact, they state that the ‘phrase [predictive policing] has broadened to include activities – such as facial recognition or “predicting” that certain individuals will commit crimes – with which we are not aligned; even the use of the word “predictive” itself does not accurately describe our business’ (Geolitica 2021). Rather, Geolitica now intends to ‘conduct risk assessments, using what-where-when historical crime data to identify the highest-risk locations of specified crimes by time of day and day of week’ (Geolitica 2021).

Thus, contextual reference (location and time) is now presented as the main focus of the tool, which – they explicitly state – lies not in the ‘predictive side’ but in ‘patrol operations management’, which should increase efficiency in keeping the community safe. From PredPol’s predictive policing approach, Geolitica moves to what is referred to elsewhere as ‘hotspot policing’ (Geolitica 2023b), applying data-driven policing with the goal to reduce crime not only through place-based policing, but also through improved community engagement (Geolitica 2023a). This aim, which is exactly the opposite of abstract policing as described in the current debate presented above, is nevertheless pursued with data-driven, algorithmic mediated strategies – that is, with increased digitisation of police activity.

As the case of PredPol-Geolitica shows, the use of algorithmic techniques does not necessarily imply lack of local reference – in fact, it may go in just the opposite direction. This tendency is confirmed by the proposal of the notion of ‘precision policing’ (Bratton and Murad 2018, Bratton 2022, Haberman 2022) for the contextualisation of algorithmic geo-analyses and corresponding prevention approaches. Also in this case, the starting point is an abstract computational technique, the NYPD’s crime-mapping and management tool CompStat (e.g. Walsh and Vito 2004, Eterno and Silverman 2010), which is used, however, not only to increase the number of arrests, but also to build and intensify strategic relationships inside the community (Lin and Rejniak 2017). The ‘precise’ focus is not only on pursuing criminals or potential criminals, but on neighbourhood policing and the attempt to build community trust in police officers. The basic idea is that police should focus their resources on those people and places, which are most closely connected to crime risks. In New York, this shift meant to change the operational strategy from stop and frisk to ‘people and geography’ (Lin and Rejniak 2017, p. 255). Precision policing hence works by ‘embracing local culture, history, environment, geography, size, demographics, and politics’ (Bratton and Murad 2018). Following this concept, the potential of technology should be used to implement more robust community engagement and prevention strategies focused precisely on the factors driving crime – in the areas where crime risk is higher, generally based on the idea that crime tends to focus in certain areas and among a minority of the people, commonly in an 80–20 ratio – 20 percent of the people experience 80 percent of all crime (Edwards 2017, p. 457, see also Weisburd *et al.* 2017).

Although precision policing is not intended to be an analytical concept but is rather understood as a political-discursive notion to shape the development of police in the U.S., we propose to take it as a reference in the empirical analysis of algorithmic policing. In fact, due also to the current austerity policy, there is a constant effort to do more with less (Beck and McCue 2009), resulting in additional efforts to focus police resources. The connection of algorithmic policing and precision policing is also confirmed by the acquisition by the police software manufacturer SoundThinking (formerly Shotspotter) of parts of Geolitica in fall 2023 to integrate its prediction technology into their system ‘ResourceRouter’ (Mehrotra and Cameron 2023) – marketed as part of their ‘SafetySmart Platform’, whose ‘foundation’ is ‘the precision policing philosophy’ (Dailly 2022).

## Case study: spatial analysis unit in a German land office of criminal investigation

To test the relationship between abstractness and precision of police work, we analysed some of the data we collected in our empirical study exploring the use of digital tools of a specific police unit in Germany. In this section, we present our object of investigation and the methodological approach we used in our analysis in more detail, before turning to the analysis itself in the subsequent section.

### Object of investigation

We observed the work of a Spatial Analysis Unit in a German Land Office of Criminal Investigation (LKA [*Landeskriminalamt*]), consisting of different geo-analytical projects targeting spatio-temporal patterns and correlations of crime in order to develop new crime prevention and investigation approaches for the local police forces.^2^ Although not all projects use machine learning algorithms, algorithmic analysis techniques play a considerable role in every project of the unit (I10).^3^ The overall goal is crime prevention, and the idea of prediction is present in different ways in many of the projects, as described below. Although only some of the projects fall under the label of predictive policing, the entire organisational unit is named after the predictive policing tool developed by the LKA since the middle of the last decade (D4). The organisational unit is now investigating further possibilities and approaches to generate spatio-temporal analysis of crime events to be implemented in police work, for example with reference to bicycle theft (I2; I19). Other kinds of future-related approaches are also studied and developed, varying in the spatial and temporal dimensions but also with reference to the kind of action induced by algorithmic results (I1; I17; I18). The unit has evolved from the original project, and predictive policing in the narrow sense can be regarded as door opener for this kind of broader future-related analysis (I1; FN4).

The organisational unit we studied is a quite special, particularly innovative unit among German police departments and institutions. Its staff consists of police officers as well as academics (especially data scientists), whereas also some of the police officers have completed academic training (FN1; I1). Many of the scientists spent a lot of their training time on algorithmic systems and the possibilities of analysing large data sets, often with a decided spatial focus (e.g. geography) (FN2; I1; I5). The working environment of the unit is unusual for the German police, as the unit cultivates a rather experimental approach, carrying out comparatively uncertain projects and pursuing open questions, which can often end in failure – which is accepted or even appreciated by superiors. This openness to error-prone research is rare in (German) police (Seidensticker 2019, Barthel 2020). Normally, the results of police-internal or police-related research are so politically relevant that they are only allowed to fail to a limited extent (see also Ritsert 2023).

The original goal of our empirical study was a focused analysis of the generation and implementation of crime predictions in the given Police Unit. We chose this special unit because its prediction approach can be considered the most sophisticated one in German police forces, and because it is based on machine learning algorithms. Moreover, the crime prediction software used in this unit is a proprietary development of the state criminal investigation office, which allowed us to gain deeper insight into the algorithmic system and in the possible further developments of the predictive approach than would have been the case with a private-sector algorithm. While at the beginning we were mainly focused on the predictive policing approach in a narrower sense, in the course of the study it became clear that the geoanalytical, data-driven approach to crime prevention involves the organisational unit as a whole, where algorithms are used in different ways in order to increase the effectiveness and efficiency of police work.

The organisation unit we studied is part of the higher-level local police authority, the Land Criminal Police Office (*Landeskriminalamt* [LKA]). One of its tasks is to conduct practice-oriented research on police topics and develop digital tools and approaches for the local police forces. Several dozen district police authorities are located under its jurisdiction. As a rule, each district police authority is responsible for the territory of a district or an independent city. According to the size of the district and its population density, the local district police authorities include several local police stations in which patrol officers are stationed. These patrol officers, called protection police (*Schutzpolizei*), have the task to ensure the maintenance of public safety and order within the assigned protection area (danger prevention). They must be distinguished from the criminal investigation department (*Kriminalpolizei*), which is also stationed in many local stations and is primarily concerned with the prosecution and prevention of criminal offences, (e.g. Groß 2006, Schulte 2017, Dosdall 2023).

### Methodological approach

In our initial analysis of the predictive policing approach conducted in the organisational unit we followed a multi-method, qualitative research design aiming at tracing the predictions from the ‘laboratory’ (the unit in the LKA) to the ‘street’ (the local police stations). We observed the creation process of the predictions in the LKA, their transfer to the analysis teams of the district police authorities and from there to the street, that is to the enaction of the predictions by local police officers (Egbert and Heimstädt forthcoming).

We were authorised by the responsible Ministry of the Interior to examine the work in the organisational unit with reference to the prediction making process and to study the assessment and implementation of the predictions in three district police authorities. We collected data using a combination of guided as well as ethnographic interviews and participant observations. Over a period of about 12 months, we spent several days at the geo-analysis unit, in the analysis departments of the district police authorities, and with patrol officers in the local police stations. In total, we spent around 75 h in the field. Field protocols of our visits were written from memory as soon as possible. Moreover, we conducted 47 interviews with members of the authorities involved and with relevant experts and practitioners from other police authorities in Germany. In total, we recorded 36 h of interview material. The audio files were transcribed verbatim. We also analysed relevant documents, mainly operational documents from the observed police departments and stations, like prediction maps and spreadsheets with the number of predictive policing missions in the past, but also project material of the geo-analysis unit of the LKA. We collected and analysed 67 documents in total.

The documents, the field protocols as well as the transcripts of the interviews were closely studied relying on a qualitative constant analysis approach as described by Kuckartz and Rädiker (2023), which is characterised in particular by a combination of deductive and inductive codes and is compatible with a grounded theory-style research process, like the one we did. Namely, the data collection and the (preliminary) data analysis took place in constant alternation (Charmaz 2014). This procedure enabled us to approach the data and the projects, themes, phenomena etc. discovered in the field with sufficient specificity with regard to our research question and at the same time with the necessary openness.

The argumentation central to this paper, for example, has developed throughout the data collection process and has informed the data collection accordingly in the further course, especially in order to find out more details about further relevant projects referring to community engagement. In course of our study of the work of the geo-analysis unit, our focus on the predictive policing application gave way to discussions and observations of the other projects carried out there. The analysis presented in the following section is a result of this broadened perspective.

## Empirical analysis

To illustrate our argument about the connection between abstract police and the use of algorithms, we selected three projects carried out by the police unit we studied that highlight on different dimensions how algorithmic police work can give rise to more geographically and temporally nuanced and, at the same time, more intense engagement with the citizens. We specifically investigate (1) the predictive policing software that gave rise to the organisation unit as a whole, (2) a project aimed at micro segment analyses for problem-oriented policing, (3) a data-based risk analysis approach for ATM blasting.

### (1) Place-based predictive policing software

The Spatial Analysis Unit we investigated was initiated on the basis of a self-developed predictive policing tool, one of the few predictive policing software still in use in German police (I1). This durability is probably due to the fact that this tool is the most complex police predictive software in the German-speaking world, internally implemented and maintained in the agency itself, that permanently employed data scientists instead of buying the required competences from outside (D4). The software operates with a machine learning model based on a random forest architecture – whose complexity has no equivalent in German-speaking countries (I5; I6; FN2). It does not only generate forecasts based on police's own data (e.g. on locations and times of burglaries), but also draws on external data such as socio-economic data on the spatial distribution of household incomes (I5; D4). The predictions refer to residential quarters, consisting on average of around 400 households, and are valid for one week (I1; D4; FN1).

The software predicts every week the risk of a domestic burglary in each residential quarter in the given federal state. The risk is assessed on the basis of a prediction score between 1 and 100 (which is not to be confused with a probability value)^4^, and only the predictive scores classified by the algorith as sufficiently reliable are communicated to the local police departments (I5; I6; I15). There, they are assessed by the respective local analysis units and forwarded to the relevant patrol forces, unless there are reasons to the contrary (such as surveillance operations in the concerned neighbourhoods or large-scale operations that tie up the available units) (I1; FN 1; FN8; FN 10). The local units are relatively free in the way they implement the predictions – which could also mean that they do not implement the predictions at all, as actually is often the case (e.g. FN9; FN 12; FN 15; FN 17; F18). In order to increase the acceptance of the local forces and avoid the impression that their daily work is being controlled with the software, in fact, nearly no concrete specifications are centrally made about the local implementation (FN 1; FN 2; I1; I20). As a result, the predictions are employed quite differently among the local departments.

In the literature, predictive policing is often discussed as a rather superficial and not very sustainable approach to prevention (Wilson 2017, Brayne 2021, Egbert and Leese 2021), because the main operational aim is understood as the use of the forecasts to send more patrols into predicted risk areas where they are supposed to deter (or displace) inclined perpetrators – without any commitment to dealing with the underlying causes and thus to real problem solving. Our empirical material, however, shows that other preventive strategies are possible. For example, in one of the district police departments we observed a rather diverse portfolio of possible reaction strategies to predictions, regularly used in its full breadth (I38; I42; FN 25). Besides the classical response of increased patrol presence in the predicted risk areas, these strategies include checkpoints of on-call police (*Bereitschaftspolizei*), also called riot police, with regard especially to relevant vehicles or persons (e.g. vans with Eastern European licence plates) (I39); measures by the crime prevention and victim protection department (e.g. information stands with the ‘prevention mobile’ on marketplaces) (I41) and actions from the relevant district service police officers (FN 24; I43; D32; D33). Whereas the members of the crime prevention and victim protection department are specialised in prevention measures focusing on the perspective of (possible) victims, the district service officers have predominantly a geographic reference with permanent assigned spaces they are familiar with. Accordingly, they take on a role similar to the neighbourhood officers active in community policing (e.g. Skogan 2008, Terpstra *et al.* 2014).^5^ On the basis of the predictions, they for example walk through the predicted risk areas and talk to the people there about the impending danger, e.g. by asking them to report suspicious observations and take appropriate security measures (close doors and windows etc.) (I43). The members of the crime prevention and victim protection department are less connected to the respective areas and the citizens there, but they also have the task to pass on to the citizens their knowledge about criminal behaviour and ways to preventing it, for example by giving information about proper security measures in houses or flats (I41; FN 24). For example, in their ‘prevention mobile’ they keep material examples of burglar-proof windows and various types of security locks (D38; D40; D41).

Although the core tasks of the district service police officers and the officers from the crime prevention and victim protection department are different, both parties and their integration in the predictive policing approach illustrate that the use of algorithmic procedures by the police does not necessarily mean that police work becomes more abstract – that is, more distant from the community and more centralised in the sense of less local engagement. Although the underlying prediction analytics are unquestionably abstract, this example ultimately shows that the form and mode of the concrete utilisation of algorithmically generated knowledge by the police is crucial to the question whether digitalised policing implies increasingly abstractly operating police force.^6^ Our results ultimately also support the analyses of Kaufmann (2018, 2019), Brayne (2021), Sandhu and Fussey (2021) as well as Egbert and Heimstädt (forthcoming), who showed that police officers discretion is still an important factor in the day-to-day implementation and construction of crime predictions. In a similar vein, Ferguson (2019, pp. 500–503) connects community policing with the predictive policing software HunchLab – which was also acquired by SoundThinking in the fall of 2018, then under the name Shotspotter (ShotSpotter 2018, Cheetham 2019) –, which offers reaction to crime predictions that are not limited to ‘short-term hot-spot solutions’ but can also include community-based strategies (Ferguson 2019, p. 501). These findings, on the other hand, contradict other analyses arguing that the increasing algorithmization of police tends to reduce the emphasis on professional police work, especially on discretionary judgment and the focus on crime prevention (McGuire 2021, Gundhus *et al.* 2022, 2023).

### (2) Micro segment analyses for problem-oriented policing

In addition to the development of the actual prediction software, another project at the geo-analysis unit analysed to which extent it was possible to reduce the geographical focus of predictions (D4; D13). As described above, the crime predictions delivered by the software we studied refer to residential neighbourhoods consisting on average of around 400 households. Although smaller than entire police districts, they are nevertheless quite wide – especially when the aim is to patrol them more intensively for up to seven days and without further specifying the relevant time of day. It is not surprising that in many cases – especially in urban police stations where the crime rate is above average – police officers do not have the time to patrol the forecast areas sufficiently often. It would therefore make sense to further narrow down the size of the forecast areas, i.e. to take a closer look at individual street sections.

The corresponding project rested on the founding idea of place-based criminology that a high proportion of overall crime takes place in a small portion of a city (Weisburd *et al.* 2017). Therefore, the police are well advised to concentrate their (scarce) resources primarily on these very neighbourhoods, i.e. on ‘street segments’ (Weisburd *et al.* 2012) located at the ‘micro geographical level’ (Weisburd *et al.* 2020). Following this insight, members of the LKA team conducted a study based on a file analysis of domestic burglaries in order to check if a relevant policing approach can be inferred: ‘By attributing crime to place-based factors, this research approach enables the police as well as other crime prevention actors to focus their efforts precisely on one place’. (D13, p. 26) They focused on domestic burglaries because this offence type was also the reference for their predictive policing approach. Domestic burglaries, moreover, show to a great degree the existence of the repeat victimisation pattern, meaning that certain locations are experiencing recurrent burglaries (Johnson 2008) – which might suggest that they have certain (place-based) characteristics making them particularly attractive to offenders. Especially in the context of crime prevention, the identification of these characteristics is considered highly relevant (D13, p. 27).

For the city analysed in this sub-project, it was found that only 3,86% of all micro segments (14,869 in total) account for half of all domestic burglary offences between 2012 and 2016. The idea from Weisburd and colleagues, therefore, was considered applicable also to the federal state in general (D13, p. 31).

Based on these results, in a next step of the micro place-based crime prevention approach a more sophisticated method was developed to deal with crime problems in the identified hot spots (D9; D12; I10; FN4). It comprised a more complex analysis of the crime problem, including algorithmic techniques. The corresponding prevention approach was much more oriented towards the root causes of the identified crime problem than in the case of predictive policing (I10; FN4).

This orientation toward problem-oriented policing (Goldstein 1979) implies that local communities are an important information source and reference for developing and enacting problem-solving strategies. The goal of the micro segment analysis project is namely to work closely with local residents – like shop owners, social workers, church leaders and other community representatives – and with other municipal departments – especially the public order office or the municipal cleansing department – in order to identify the probable sources of the concentration of crime in certain micro segments (D12; I10; FN4). The implementation of possible problem-solving strategies is also done in close relationship with the local stakeholders. Overall, the resulting approach is contextual and personal, although it relies on abstract algorithmic procedures.

A very similar project is being developed by the federal criminal police office (BKA [*Bundeskriminalamt*]) together with the city of Gelsenkirchen in the German federal state North Rhine-Westphalia, where an ‘Interdisciplinary early warning system’ working with the tool ELSA (*evidenzbasierte lokale Sicherheitsanalysen* [Evidence-based local safety analyses]) has been implemented (Stadt Gelsenkirchen 2022). ELSA queries the factors influencing local security in eight modules on topics such as crime, order, crime prevention, cooperation between authorities, resources, infrastructure, economy, and demography. The data recorded, such as statistics and public enquiries, is for the most part already available to the local authorities. In addition, staff surveys are conducted. After the data has been collected and evaluated, the results are visualised with a traffic light system (Mayer 2021) (D61; D62; D63). Here again we can identify the combination of a complex and highly abstract analysis tool with a community-focused, personal, and contextual prevention approach.

### (3) Risk analysis of ATM blasting

A third relevant project we observed in our ethnographic study of the LKA-geo-analysis unit refers to one of the currently most important offence types in terms of publicity and political relevance in Germany: the blowing up of ATMs (Klenner and Lang 2023) (D56). Since a few years the number of ATM blastings is significantly rising, putting increasing pressure on politics and police authorities (Bartsch *et al.* 2023). The police generally assume that this development originates mainly from Germany’s western neighbours, especially the Netherlands (FN29; I17; I46). Most of the perpetrators residing there have highly professional structures. This is why it is considered to be so difficult to solve the underlying crime problem and why the success of investigations has been limited so far (I46). In addition, the perpetrators are characterised by a modus operandi that makes it difficult for the police to arrest them or prevent them from fleeing to neighbouring countries (I17; FN29). As a rule, the perpetrators use the well-developed motorways to escape in their highly motorised cars that are often too fast for police cars. Attempts to hinder them can only be carried out at great risk to police officers and other road users, which is why suitable arrest tactics are rare (I46; FN29).

The drivers behind this development are on the one hand seen in the fact that in the Netherlands there are significantly fewer ATMs and a systematic cooperation within the banking industry, resulting in higher security level of the devices (I17). On the other hand, in Germany the number of ATMs is very high, also in rural areas, and these cash machines are often not sufficiently well secured (I46). The banks refuse to significantly enhance the security level of their ATMs because they are well insured and do not see the economic necessity (FN29). In fact, from a cost–benefit logic, it is cheaper for the banks to leave the security standards of the ATMs low and accept the blasts. For the police and the relevant politicians, however, such a situation is not acceptable because third parties (other road users, residents, etc.) are also affected by the explosions (Neuerer and Atzler 2023) (FN29; I17).

An important element of the strategy recently developed by the police to decrease the number of ATM blastings is the close cooperation with the banks to, on the one hand, convince them to invest more in the security of their machines, and, on the other hand, obtain information about the individual machines and their locations. In Hesse, for example, the ‘alliance ATMs’ (*Allianz Geldautomaten*) was founded (Hessisches Ministerium des Innern und für Sport 2023) to improve the cooperation between police and banks. In the ATM blasting risk classification case we studied, the analysis team sent questionnaires to all banks operating in the respective federal state to get information about the number and places of all their ATMs plus information about existing security characteristics (e.g. is there gluing or colouring technology? How is the building secured? Is there a night lock? Is there an alarm system?) (I17; FN5; FN6).

The goal of this collaboration is to reduce the opportunities for crime, by convincing the banks to enhance their security measures for cash machines. But it is also the goal to get information about their ATMs in order to determine a risk classification of each cash machine, enabling more targeted prevention as well as response and search strategies (I17). The unit we studied developed an analysis system relying on the risk terrain modelling technique used also in predictive policing (Caplan and Kennedy 2016, Rummens and Hardyns 2020, Hälterlein 2021). It is based on a geographical model that works with bands placed over the map of the state in question, each containing the same number of ATMs (with narrower bands when there is a high density of ATMs, as in large cities). As the police assumes that the shorter the distance to the Western neighbour countries, the higher is the blasting risk, the bands are evaluated according to their distance to the Netherlands and combined with information on the respective ATMs to calculate a risk category for each cash machine (I17; FN6). Three risk categories are used: high risk (red), medium risk (yellow), low risk (green). A high risk ATM, for example, is located far to the west in the state and poorly secured, e.g. does not contain ink cartridges that would render the money unusable in case of an explosion, and is also accessible at night. The idea is that members of the local crime prevention and victim protection department, also referred to as ‘technical consultants’, will visit high-risk sites and clarify if the classification makes sense (i.e. whether there are obvious reasons against a high-risk assessment). If necessary, further measures will be discussed with the local responsible persons of the banks (I17).

In this ATM blasting risk modelling-project, we observe again a remarkable combination of an abstract technological approach to estimate risks via an algorithmic mediated risk analysis technique with a preventive strategy executed in close relationship with the relevant local actors. The goal is to generate precise knowledge about the geographic distribution of risks in order to enhance policing activities.

## Conclusion: from abstract police to precision policing

The growing digitisation in our society unquestionably also affects policing, which makes use of increasingly refined IT tools (Edwards 2017). And these tools certainly adopt abstract technologies, which operate with notional numbers and are often non-transparent, especially when they rely on machine learning technologies. But the abstraction of technology, we argue, does not necessarily entail an increase in abstraction of police work. This paper contrasts the ‘abstract police’ debate with an analysis of police practices that use digital technologies to achieve greater precision. While the notion of abstract policing assumes that computerisation distances police officers from their community, our empirical study shows that the adoption of abstract procedures does not by itself imply a detachment from local reference and community contact. What we call contextual reference can be productively combined with the impersonality and anonymity of algorithmic procedures, leading to more effective and focused forms of collaboration with local entities. Our results ultimately also support the existing analyses which highlight that police officers’ discretion continues to be relevant in algorithmic policing, possibly leading to an innovative form of ‘reflexive securitization’ (Edwards 2017, p. 459).

Our empirical investigation of a geo-analysis unit in a German Land Office of Criminal Investigation comprises three projects. The first is the use of a complex predictive policing software to forecast the risk of domestic burglaries. The practical implementation of the tool has led not so much to using the software for the suppression of possible perpetrators (an activity that turned out to be quite unfeasible, as well as questionable) but rather to intensifying contacts with the community and making preventive measures more effective. The second project we analysed pursues an attempt to reduce the extent of the areas targeted by the prediction, aiming at a more fine-grained spatial analysis. In this case, the use of algorithmic techniques made possible a more complex and precise analysis of crime problems, oriented to root causes rather than abstract deterrence. The third project is focused on the problem of increasingly frequent ATM blastings. In this case the police, using highly abstract methods of risk terrain modelling, established a close collaboration with local stakeholders.

On the basis of our empirical results, we suggest a more nuanced understanding of the digitalisation of police work, centred on precision rather than abstractness. Many digital tools developed and implemented in policing, although being fundamentally abstract, tend to enhance the ability to spatiotemporally differentiate crime risks and to prioritise policing areas and strategies accordingly. This trend can also involve police projects implying a closer collaboration with the community as well as a more locally sensitised approach by the police. Thus, rather than leading to a progressive estrangement from the community of reference, as is often feared, the use of digital techniques can enable experimentation with innovative forms of precision policing, particularly in the field of prevention.

## Notes

1 Predictive Policing as a threat and/or challenge for professional identity is also discussed by Brayne (2021, p. 77) and Wilson (2017, p. 118). See also Sandhu and Fussey (2021) on the reluctancy of police officers to use crime prediction software.

2 The empirical study was conducted at Bielefeld University, Germany, as part of the ERC-advanced grant project ‘The Social Consequences of Algorithmic Forecast in Insurance, Medicine and Policing’ (Grant agreement No. 833749).

3 References to the contents of our empirical data are indicated by the type of material (I = interview; D = document; FN = fieldnotes) and the respective number.

4 A forecast value of 70 does not mean that the probability of a burglary in the area in question is 70%. In reality, it is much lower.

5 An overview about the (non-)adoption of community policing in Germany is given by Feltes (2013; 2014).

6 This is even more remarkable in the case of predictive policing, since this policing strategy aims at ‘doing more with less’ (Beck and McCue 2009), implying an organisational logic originating from new public management – which Terpstra et al. connected with abstract police (Terpstra *et al.* 2019).
